# Rapid and sensitive detection of tenuazonic acid in agricultural by-products based on gold nano-flower lateral flow

**DOI:** 10.3389/fbioe.2022.1021758

**Published:** 2022-10-07

**Authors:** Peiyuan Cai, Rongzhi Wang, Sumei Ling, Shihua Wang

**Affiliations:** ^1^ State Key Laboratory of Ecological Pest Control for Fujian and Taiwan Crops, Key Laboratory of Pathogenic Fungi and Mycotoxins of Fujian Province, School of Life Sciences, Fujian Agriculture and Forestry University, Fuzhou, China; ^2^ College of Chemistry, Chemical Engineering and Environment, Minnan Normal University, Zhangzhou, China

**Keywords:** test strip, lateral flow immunochromatographic assay, colloidal gold, multi-branched gold nano-flower particle, mycotoxin, TA

## Abstract

Tenuazonic acid (TA) is a highly toxic mycotoxin mainly generated by the fungi of *Alternaria* genus and widely contaminates agricultural by-products. Given the threat of TA to food-security, it is very important to develop rapid and sensitive detection methods for TA monitoring. In this study, gold nano-particles (AuNP) with average diameter near 17.25 nm were prepared, and the developed AuNP-based strip has an assay time of 15 min with visual limit of detection (LOD) of 12.5 ng/ml and threshold of 100 ng/ml. To further improve sensitivity, multi-branched gold nano-flowers (AuNF) with average diameter near 50 nm were prepared and characterized by UV-VIS and TEM, and the established AuNF-based strip has visual LOD of 0.78 ng/ml and threshold of 50 ng/ml within 15 min. Both assays were applied to determine TA in apple juice and tomato ketchup, and the results were consistent with that of UHPLC-MS/MS. Thus, these assays could be applied for rapid determination of trace TA in real samples.

## Highlights

- This AuNP strip test had visual LOD of 12.5 ng/ml and threshold of 100 ng/ml.
- This AuNF strip test had visual LOD of 0.78 ng/ml and threshold of 50 ng/ml.
- This AuNF achieved the sensitivity for 16-fold better than that of AuNP.

## Introduction

The contamination of mycotoxins in foodstuff has turned into a global issue, and an appalling reality of near twenty-five percent of worldwide contamination of the agricultural products with mycotoxins was reported by the Food and Agriculture Organization estimate (Eskola et al., 2020). Tenuazonic acid (TA) is a toxic mycotoxin which is mainly generated by the fungi of *Alternaria* genus (Gross et al., 2011). TA has been confirmed to have strong acute toxicity and chronic toxicity, potential cytotoxicity and even oncogenicity (Asam et al., 2011). The chronic exposure to TA could cause tissue hemorrhage, motor dysfunction, circulatory collapse, severe dysplasia and even serious shock and death for several animals (Yekeler et al., 2001; Ostry, 2008; Zhou and Qiang, 2008). During low doses administration at the level of 10 μg/g of feed for 2 or 3 weeks, TA could result in tissue lesions of young chicken (Giambrone et al., 1978). The half lethal dose (LD_50_) of TA to chicken embryos was 548 μg per egg (Griffin and Chu, 1983), and the LD_50_ to brine shrimp larvae was 75 μg/ml (Panigrahi and Dallin, 1994). Moreover, TA has already been listed in the toxic chemicals of the United States Food and Drug Administration (FDA) for its high toxicity (Wang et al., 2021a). Unfortunately, TA widely contaminates food and feed, especially vegetables, fruits and their products, and even the positive rate of TA from vegetables and fruits related by-products were reported achieving one-hundred percent in some individual region (Ostry, 2008; Gross et al., 2011; Asam and Rychlik, 2013). Therefore, it is in urgent need to develop convenient, sensitive and rapid methods capable of real-time and low-cost determination for TA.

Several instrumental methods for determination of TA were established, including high performance liquid chromatography (HPLC), liquid chromatography tandem mass spectrometry (LC-MS/MS) and ultra-high performance liquid chromatography tandem mass spectrometry (UHPLC-MS/MS) (Siegel et al., 2009, 2010; Lohrey et al., 2013; Hickert et al., 2015; Puntscher et al., 2018). These instrumental methods are high-accurate, high-sensitive and good-reproducible, however, they are not adaptable for the large-scaly practical promotion due to the inseparable need of professional operation and expensive equipment (Anfossi et al., 2013; Zangheri et al., 2015). Immunoassays are practical methods which are highly specific and sensitive with low cost and easy operation, adaptable for wholesale determination (Ang et al., 2015; Yan et al., 2018). The enzyme-linked immuno-sorbent assay (ELISA) is the most common immunoassay, and a recent research reported an ELISA method for detection of TA with limit of detection (LOD) of 1.0 ng/ml and half inhibitory concentration (IC_50_) of 18.5 ng/ml (Liang et al., 2020). Recently, Wang and coworkers reported a bioluminescent enzyme immunoassay (BEIA) method with IC_50_ of 6.5 ng/ml and LOD of 0.7 ng/ml (Wang et al., 2021a). Our previous work demonstrated that the ELISA method based on anti-TA monoclonal antibody (McAb) 6D5 had LOD of 0.17 ng/ml and IC_50_ of 2.50 ng/ml (Cai et al., 2021). However, ELISA and BEIA protocols contain multiple steps of washing and incubating, which leads to a longer assay time (Ling et al., 2015). To this end, our previous work proposed a new strategy of lateral flow immunoassay (LFIA) for TA detection based on platinum-modified colloidal gold by catalyzing precipitation-type tetramethylbenzidine, which includes two steps of lateral flow and color developing, and the visual LOD was 0.39 ng/ml with the assay time of 0.5–1.0 h (Cai et al., 2021).

As representation of rapid and convenient immunoassay, LFIA of one-step process (just including lateral flow step) still has great advantages in the assay time of 5–15 min and the visual detection (Wang et al., 2017; Wu et al., 2020). LFIAs based on colloidal gold nano-particle (AuNP) probes have been devoted to detect different kinds of toxic substances for decades because of their easy production and convenient conjugation with antibodies (Ling et al., 2020; Wang et al., 2021b). Multi-branched gold nano-flower (AuNF) particles were a kind of shape-modified AuNP particles, which grew to a larger size and formed flower-shape on the surface of the AuNP seeds (Ling et al., 2020). The localized surface plasmon resonances (LSPR) of AuNF exhibited stronger optical extinction capability (Dondapati et al., 2010). Moreover, the AuNF particles had complex three-dimensional (3D) structure and presented larger specific surface benefiting for the reduction of steric hindrance during the conjugation with antibodies (Ji et al., 2015). Therefore, it could be used as an ideal signaling material to promote the sensitivity of immuno-sensors (Wang et al., 2021b; Ling et al., 2021).

Given the high contamination rate of TA and the urgent need in rapidly sensitive monitoring for food-security, highly effective methods for trace TA determination were established and improved in this study. To this end, two practical one-step LFIAs based on AuNP and AuNF were respectively developed for TA detection in agricultural by-products. Combining the naked eyes, the developed LFIA strip tests could quickly achieve the visualization for trace TA determination in real samples, while providing the high sensitivity. In present research, the sensitivity of AuNF-based LFIA was 16-fold to that of the conventional AuNP-based LFIA. Our results showed that these two kinds of LFIAs with different properties have the great potential in highly sensitive on-site determination of TA.

## Materials and methods

### Reagents and instruments

Tenuazonic acid and other common mycotoxin standards, bovine serum albumin (BSA) and goat anti-mouse IgG antibody (GAMA) were obtained from Sigma-Aldrich (MO, United States). Trisodium citrate, HAuCl_4_ 4H_2_O and hydroquinone were purchased from Macklin (Shanghai, China). Nitrocellulose (NC) membrane was provided by Jieyi BioTech. (Shanghai, China). Anti-TA antibody reagent (anti-TA McAb 6D5) was prepared in our laboratory according to our previous work (Cai et al., 2021). All the other reagents were analytical reagent grade. Ultraviolet-visible spectrum (UV-VIS) was tested by spectrophotometer (PerkinElmer, Waltham, MA, United States). Tecnai G2 F30 transmission electron microscope (TEM) (FEI, Hillsboro, OR, United States) was used to scan the diameter and shape of the prepared nanoparticles. The UHPLC-MS/MS was performed by Waters ACQUITY UPLC-XEVO TQ-S MS with ACQUITY UPLC BEH C18 (Waters, Milford, MA, United States).

### Preparation of gold nano-particles and gold nano-particles-based probe

AuNP was prepared by trisodium citrate reducing method (Ling et al., 2015). Briefly, 2 ml of 1% trisodium citrate was added into 97 ml boiling deionized water, and then 1 ml of 1% HAuCl_4_ was added rapidly into the above solution and mixed, keeping heating and stirring until the color becoming stable brilliant wine red, then cooled down to the room temperature followed by diluting to 100 ml with deionized water. The resulted AuNP was characterized by UV-VIS spectrum and TEM. Then the AuNP-based probe was prepared by the previous method (Ling et al., 2018). Briefly, 250 μl of 0.1 M K_2_CO_3_ was mixed with 10 ml AuNP to balance pH value, then 75 μl of 1.56 mg/ml anti-TA McAb 6D5 was slowly added and kept stirring gently for 1 h. The probe was centrifuged at 12,000 r/min for 20 min, and the dark-red flow layer was redissolved in 1 ml diluent.

### Construction and characterization of gold nano-particles-based test strip

TA antigen (TA-O-BSA) were prepared according to our previous research (Cai et al., 2021). The AuNP-based strip was constructed based on AuNP probe, which was composed of sample pad, conjugate pad, nitrocellulose (NC) membrane, absorbent pad and base pad, with 4 mm width. Antigen and GAMA were respectively dispensed as test (T) and control (C) lines, and probe was sprayed onto the conjugate pad. Lateral flow process was set at 15 min. Different toxins were used to assess the specificity of the AuNP-based strip (Saeed et al., 2017; Ling et al., 2018). To determine the sensitivity, different concentrations of TA were tested respectively, and then the sensitivity and visual LOD were assessed by the visual result (Kong et al., 2017; Ling et al., 2018). To evaluate the accuracy, two kinds of spiked samples (apple juice and tomato ketchup) with different concentrations of TA were pretreated (Gross et al., 2011; Cai et al., 2021), then detected by the developed assay. Finally, four random real samples from local supermarket (Fujian, China) were detected and the results were confirmed by UHPLC-MS/MS.

### Preparation of gold nano-flowers particles and gold nano-flowers-based probe

Fifty nanometers AuNF was prepared by gold seeding method with minor modification (Zhang et al., 2019; Ling et al., 2021). Briefly, 100 ml deionized water was adjusted by 5 M NaOH to pH7.0 and then 500 μl of the above AuNP was added as seeds. 750 μl of 1% HAuCl_4_ and 300 μl of 1% trisodium citrate were subsequently mixed with the above solution. Then, 1,000 μl of 30 mM fresh hydroquinone was added into the solution and mixed vigorously for 30 min, until the color becoming stable dark-blue. Finally, the resulted AuNF was characterized by UV-VIS and TEM. The optimal conditions for McAb labeling were tested by sodium chloride interference method (Chenggang et al., 2008), and the AuNF-McAb probe was synthesized as follow: 10 ml AuNF was adjusted to the optimal pH by 0.1 M K_2_CO_3_, then an optimal amount of anti-TA McAb was added dropwise. The mixture was stirred gently in ice bath for 1 h. Then, PEG20000 was added at the final concentration of 1% and kept stirring for 45 min, and then BSA was added at the final concentration of 1% for stirring another 45 min. The probe was centrifuged at 6,500 r/min for 20 min, and then the dark-blue flow layer was redissolved in 1 ml of the AuNF probe diluent (basic formula: 1% BSA +0.5% PEG20000 + 5% Trehalose +0.5% Tween20 dissolved in 0.01 M PBS of pH7.4).

### Construction and characterization of gold nano-flowers-based test strip

The AuNF-based test strip was consisted of base pad (DB-6), NC membrane, sample pad, conjugate pad and absorbent pad. Conjugate pads and sample pads were blocked by the buffer (formula: 1% BSA + 0.5% PEG20000 + 0.5% Tween dissolved in 0.01 M PBS of pH7.4) at 37°C for 60 min, then dried at 37°C for overnight. Antigen and GAMA were respectively dispensed with 1.5 μl/cm as T line and C line, then the NC membrane was dried at 37°C for 24 h. The AuNF-McAb probe was sprayed onto conjugate pad and then dried in vacuum. Finally, NC membrane, sample pad, conjugate pad and absorbent pad were successively installed on the base pad, and then cut into 4 mm width. To perform the AuNF-based assay, 100 μl of analyte liquid was allowed to lateral flow through the T and C lines, and be trapped based on the immunoreaction. The time of the lateral flow process was set at 15 min. Finally, the characterization and identification of the AuNF-based strip assay were performed according to the same protocols to the developed AuNP-based strip test.

### Tenuazonic acid analysis in sample

The samples were pretreated according to the previous protocol with minor modification (Gross et al., 2011; Cai et al., 2021). For the detection of lateral flow assay, the pretreated samples were redissolved in 0.01 M PBS containing 5% methanol. For the determination of UHPLC-MS/MS, the pretreated samples were redissolved in 100% methanol. Tomato ketchup and apple juice samples without containing TA were obtained from local supermarket, and different concentrations of TA standard were spiked into these two samples and then pretreated for the detection by the prepared LFIAs. Four random samples from local supermarket (Fujian, China) were detected as real samples by the strip tests. UHPLC-MS/MS was performed by the gradient elution method according to our previous work (Cai et al., 2021), and applied to verify the test results of the strips assays.

## Results

### Identification of the gold nano-particles and gold nano-particles-based probe

**FIGURE 1:**
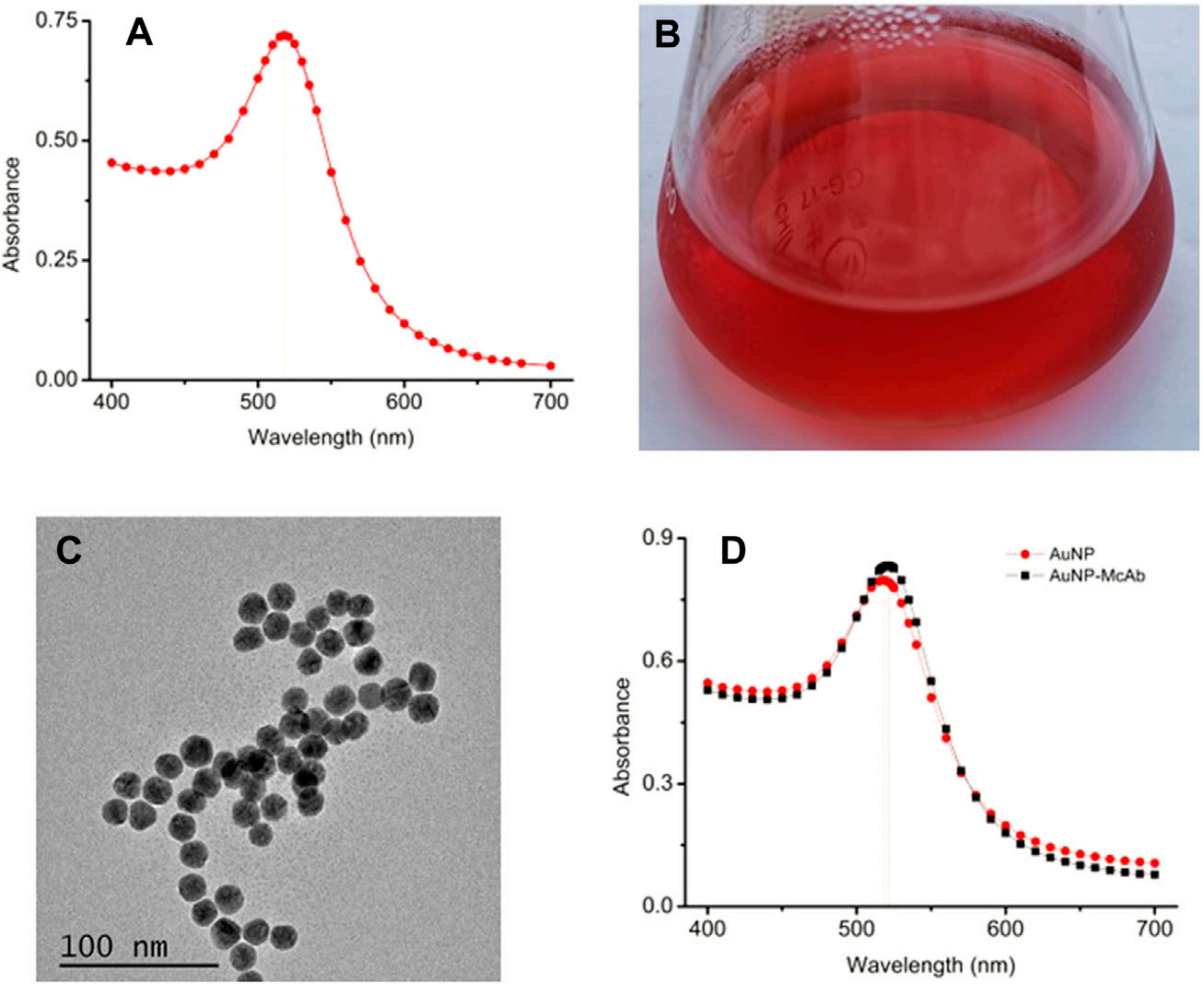
Preparation and identification of gold nano-particles (AuNP). **(A)** UV-VIS spectrum scanning of AuNP. **(B)** The prepared AuNP solution. **(C)** TEM image of the AuNP. **(D)** UV-VIS spectrum of the AuNP-McAb conjugate.

The AuNP were synthesized for the construction of the AuNP-based test strip, meanwhile, they were also used as the seeds for AuNF particles. Therefore, the size, morphology and especially the quality of the AuNP would greatly affect the AuNF. In this study, the AuNP was produced by trisodium citrate reducing method (Ling et al., 2015), and the results from Figure 1A showed that the prepared AuNP had a UV-VIS peak at 519.3 nm. The result from Figure 1B showed that the prepared colloid displayed limpid without any precipitate at the bottom or any floating impurities on the surface. The TEM image from Figure 1C showed that the prepared AuNP was nearly circular with relatively uniform size and the average diameter of 17.52 nm. These results demonstrated that the prepared AuNP was suitable for subsequent experiments. The optimal conditions for AuNP labeling McAb (AuNP-McAb) were tested by sodium chloride interference method (Chenggang et al., 2008). The result demonstrated that the optimized dosage of anti-TA McAb was 1.5 μl of McAb (1.56 mg/ml) corresponding to 200 μl AuNP liquid, and the optimized pH range was 5.0 μl of 0.1 M K_2_CO_3_ corresponding 200 μl AuNP liquid. The result from Figure 1D showed that the UV-VIS peak of AuNP-McAb conjugate was red-shifted from 519.3 to 523.7 nm compared with that of AuNP, indicating that the AuNP had been successfully labeled with anti-TA McAb to construct the AuNP-based probes (López-Marzo et al., 2013; Hong et al., 2020).

### Characterization of gold nano-particles-based lateral flow immunoassay

**FIGURE 2:**
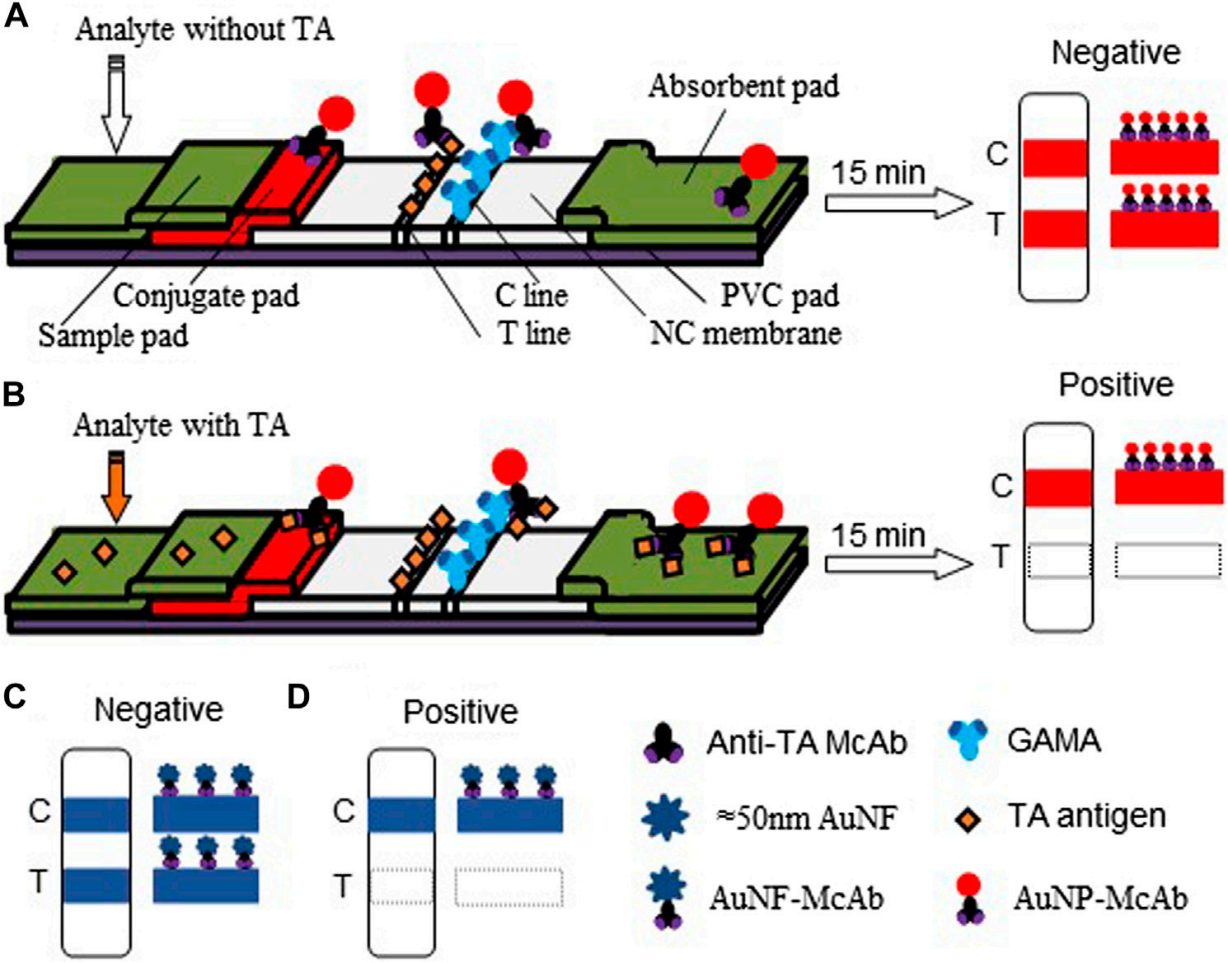
Schematic diagram of the AuNP-based and AuNF-based strip tests. **(A)** The conventional AuNP assay and the diagram of negative result. **(B)** The competition mechanism of AuNP assay and the positive result. **(C)** The construct of AuNF test strip and the negative result. **(D)** The competition mechanism under signal enhancing and the positive result.

**FIGURE 3:**
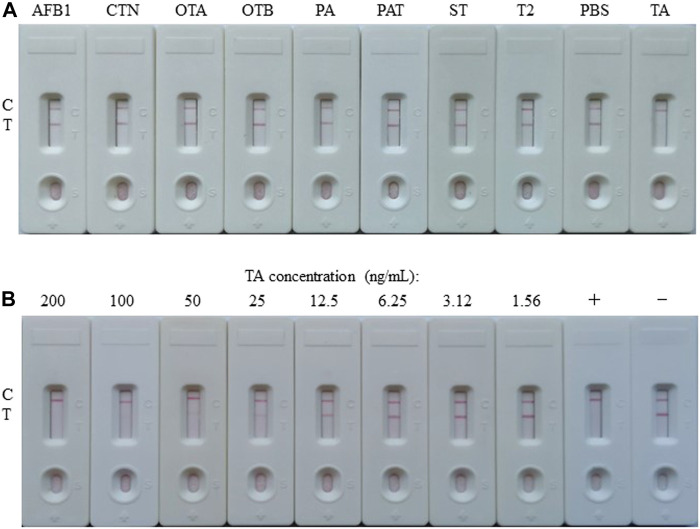
Preparation and characterization of the AuNP-based LFIA. **(A)** Specificity of the AuNP-based strip test with other toxins, such as aflatoxin B1 (AFB1), citrinin (CTN), ochratoxin A (OTA), ochratoxin B (OTB), penicillic acid (PA), patulin (PAT), sterigmatocystin (ST), or trichothecenes-2 (T2), while PBS as negative. **(B)** The sensitivity of the AuNP-based strip test. +: positive, ‑: negative.

For the construction of the AuNP-based test strip, the test line (T-line) was coated 1/4 diluted antigen (3.20 mg/ml) with 1.25 μl/cm, while the control line (C-line) was coated 1/4 diluted GAMA (1.00 mg/ml) with 1.25 μl/cm. The competitive AuNP-based test strips were constructed as shown in Figures 2A,B. While there was without TA in the analyte, many probes would be captured by the antigen (TA-O-BSA) coated at T-line and thus aggregated to form an intense band on T-line (Figure 2A). On the contrary, when there was enough free TA contained in the analyte, the probes would be captured by the free TA, which blocked the binding of probe with TA-O-BSA coated at T-line, therefore, there was no visible band emerged on the T-line (Figure 2B). The signal appearance of C-line provided and verified the validity of the strip test, otherwise invalid. The specificity of the constructed test strip was tested, and the result in Figure 3A displayed that the AuNP-based strip could specifically recognize TA, and there was no visible band on the T-line with TA spiked sample. The sensitivity was also measured, and the result in Figure 3B showed that there was no color band at the T-line when the concentration was above 100 ng/ml, and the color intensities of the T-line was still lighter than that of the C-line at the concentration of 12.5 ng/ml. Therefore, the detection threshold was 100 ng/ml and visual LOD was 12.5 ng/ml.

### Preparation and characterization of gold nano-flowers particles

**FIGURE 4:**
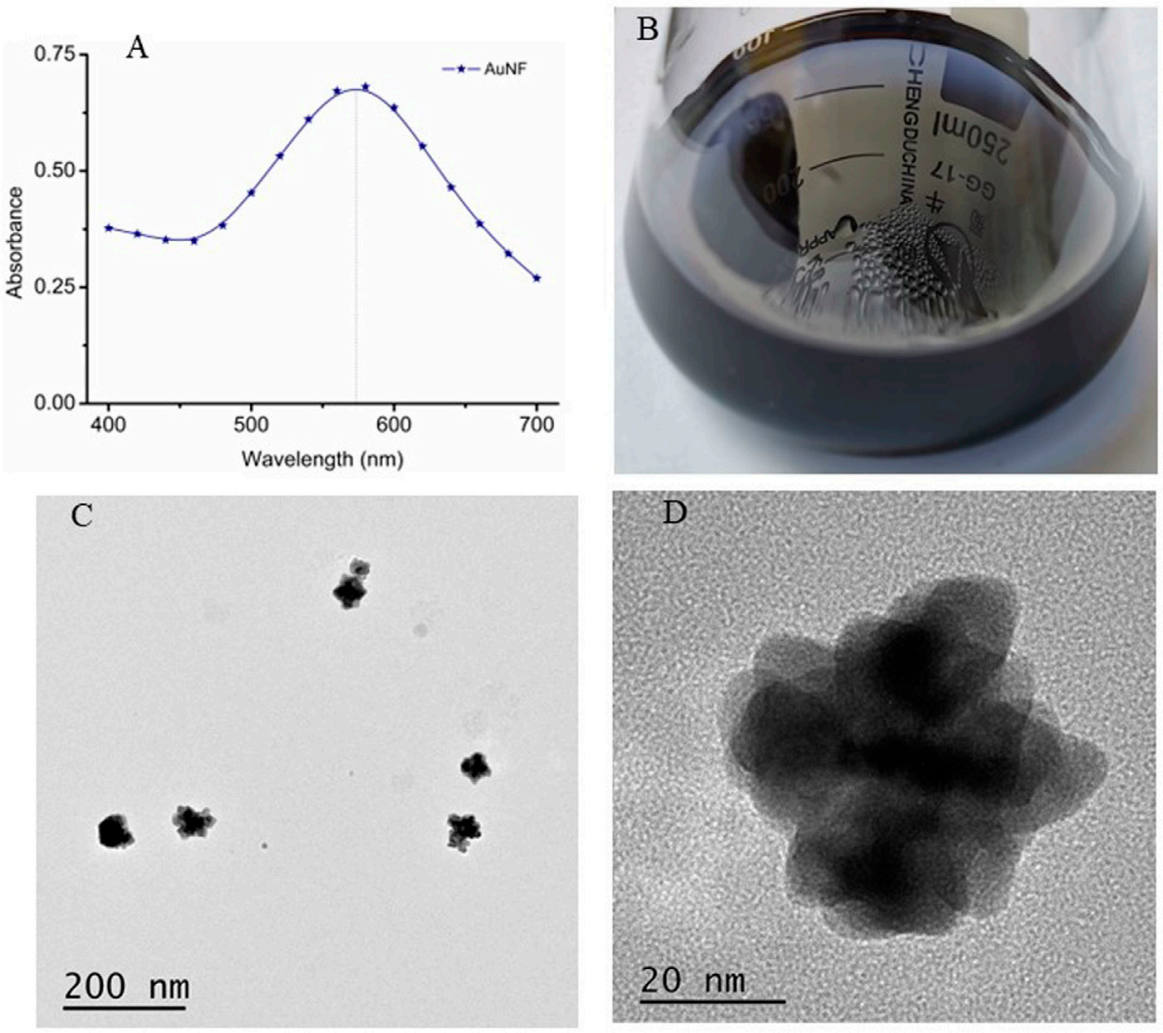
Preparation and characterization of AuNF nanoparticles. **(A)** UV-VIS spectrum scanning of AuNF. **(B)** The prepared AuNF solution. **(C)** TEM image of the AuNF at the scale of 200 nm. **(D)** TEM image of the AuNF at the scale of 20 nm.

AuNF particles have been widely applied to enhance the sensitivity of lateral flow immunoassay due to its multi-branched surface, better extinction coefficient and larger size (Ji et al., 2015; Ling et al., 2021). In this study, the AuNF particles were produced by the hydroquinone mediated seeding growth method with the 17.52 nm AuNP (Figures 1A–C) as seeds, and the prepared AuNF was identified by UV-VIS spectrum scanning. As shown in Figure 4A, the obtained AuNF had a maximum absorption wavelength near 580 nm, which belongs to the range of sizes suitable for labeling antibody (Ji et al., 2015; Wang et al., 2021b). The result from Figure 4B showed that the prepared liquid displayed transparent in dark-blue color without any precipitate of aggregation at the bottom, and the particles were dispersed well without any floating impurities on the surface of the liquid. The TEM images from Figures 4C,D demonstrated that the sizes of AuNF particles were about 50 nm of average diameter and the particles exhibited relatively uniform size and obviously multi-branched shape of the surface, rather than the circular structure of the traditional colloidal gold particles. These results demonstrated that the prepared AuNF with large size and three-dimensional multi-branched structure was available and suitable for the further construction of AuNF-based probe.

### Preparation and identification of gold nano-flowers-monoclonal antibody probe

**FIGURE 5:**
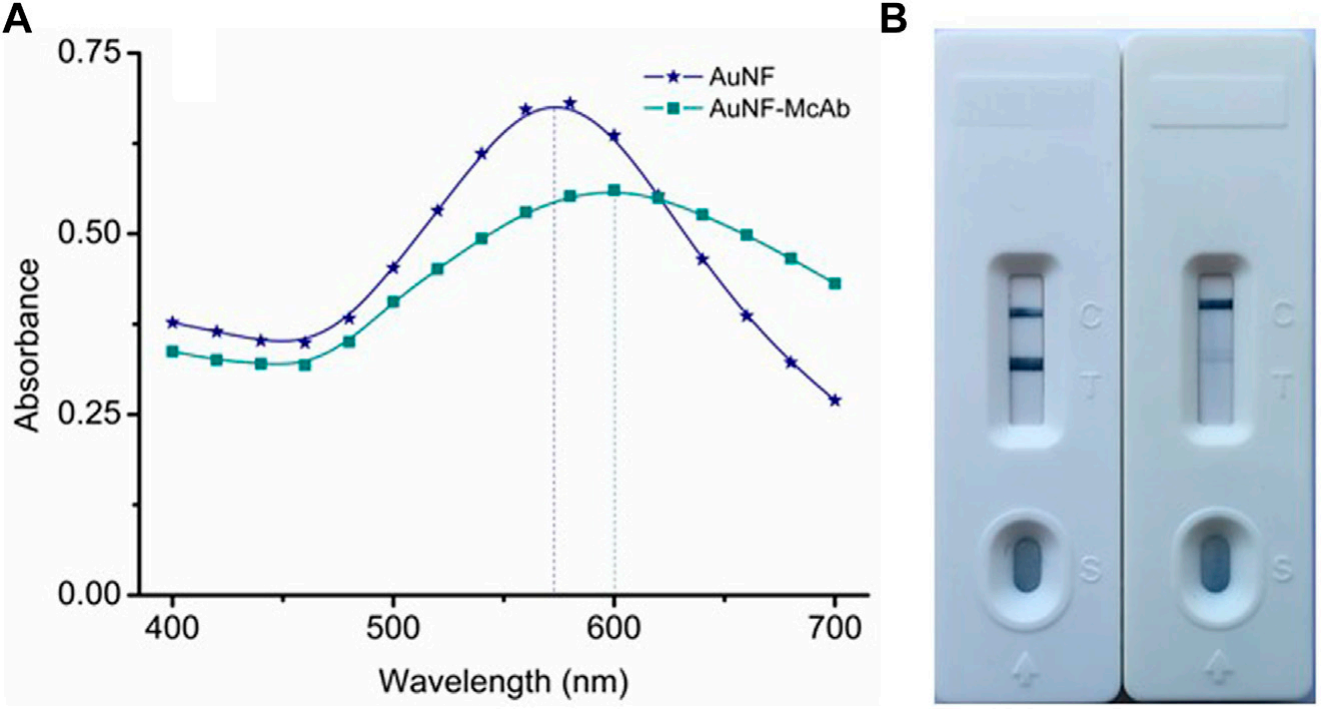
Preparation and identification of AuNF-based probe. **(A)** UV-VIS spectrum scanning of the AuNF-McAb conjugate. **(B)** The activity identification of AuNF-McAb probe.

The pH value and the McAb dosage were crucial for the preparation of the AuNF labeled McAb (AuNF-McAb) and even had an important bearing on the sensitivity of the AuNF-based LFIA (Yan et al., 2018; Ling et al., 2020). To this end, the sodium chloride interference method was performed with the same protocol of AuNP. The result of optimized McAb dosage showed that 1.5 μl of McAb (1.56 mg/ml) was added per 200 μl AuNF, and the result of optimized pH range showed that 1.0 μl of 0.1 M K_2_CO_3_ was used per 200 μl AuNF. Then the conjugation of AuNF-McAb was monitored by UV-VIS spectrum and the results were showed in Figure 5A. The UV-VIS peak of AuNF-McAb red-shifted from 580 to 600 nm compared with AuNF, demonstrating that the AuNF had been successfully conjugated to anti-TA McAb to prepare the AuNF-based probes (López-Marzo et al., 2013; Hong et al., 2020; Ling et al., 2020). The activity of the prepared probe was tested and the results were shown in Figure 5B. When PBS sample was added, the T and C lines both normally showed blue color which means a negative result. When TA spiked sample was added, the T line was disappeared, which means a positive result. These indicated that the prepared probe was active and could be used for subsequent experiments.

### Optimization of test conditions for gold nano-flowers-based strip

It was a crucial procedure to optimize the parameters for the construction of the AuNF-based LFIA strip (Yan et al., 2018; Hong et al., 2020). The evaluation criterion was based on employing lower concentration of TA target in the T-line as possible while maintaining the effectiveness of the AuNF-based test strip (Wang et al., 2021b; Ling et al., 2021). To generate T-line, the antigens of TA-O-BSA (3.20 mg/ml) were diluted with 10 mmol/ml PBS buffer according to the doubling dilution method (1/1, 1/2, 1/4, 1/8, 1/16, and 1/32), and then were respectively coated at the NC membranes with 1.5 μl/cm, followed by drying at 37°C for 24 h. The tested results showed that the dilution of 1/8 for spraying the T-line had a suitable color-density for visual judgment, and the final concentration of TA-O-BSA was 0.4 mg/ml. To generate the C-line, the GAMA (1.0 mg/ml) were optimized by the above method, and the results demonstrated that the dilution of 1/4 was the optimal dilution for generating C-line, namely, the final concentration of GAMA was 0.25 mg/ml. For spraying the conjugate pad, the obtained probes were diluted according to 1/1, 4/5, 3/5, 2/5, 1/5, and 1/10 respectively with the probe diluent, and then dried in vacuum after spraying with 2.5 μl/cm respectively. The results showed that the dilution of 2/5 was the optimal dilution for preparing conjugate pad. The formula of probe diluent was also adjusted, and the optimal diluent formulation was 0.5% BSA + 0.5% Glycine + 5% Trehalose + 0.5% Tween20 + 0.05% NaN_3_ dissolved in 0.01 M PBS buffer of pH7.4.

### Construction of gold nano-flowers-based lateral flow immunoassay

**FIGURE 6:**
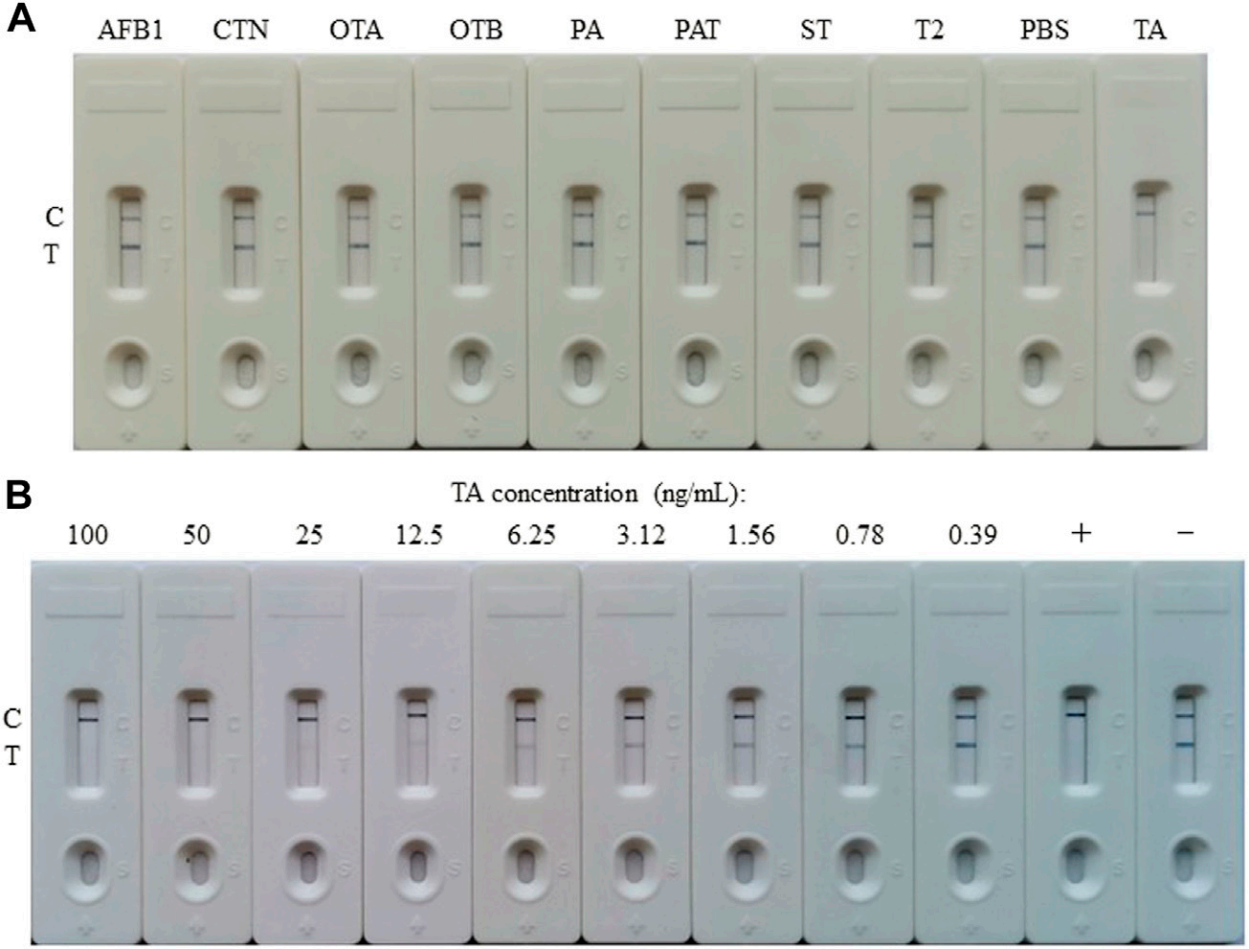
Characterization of the AuNF-based LFIA. **(A)** Cross-reactivity of AuNF-based strip test with other toxins, including aflatoxin B1 (AFB1), citrinin (CTN), ochratoxin A (OTA), ochratoxin B (OTB), penicillic acid (PA), patulin (PAT), sterigmatocystin (ST), or trichothecenes-2 (T2), while PBS as negative. **(B)** The sensitivity of the AuNF-based strip test. +: positive, ‑: negative.

The competitive schematic of the AuNF assay was demonstrated in Figures 2C,D, which is similar to conventional AuNP assay (Figures 2A,B), and the AuNF was used for signal enhancement of the detection. Compared with the conventional AuNP strip test (Figures 2A,B), the dosage of antigen (TA-O-BSA) coated at T-line and the dosage of matching probe (AuNF-McAb) on the conjugate pad could be relatively reduced due to the improved signal of AuNF (Figures 2C,D), therefore, the sensitivity of the AuNF-based assay was enhanced. To assess the feasibility of the AuNF-based strip test, the specificity was performed with a serious of common toxins, such as aflatoxin B1 (AFB1), citrinin (CTN), ochratoxin A (OTA), ochratoxin B (OTB), penicillic acid (PA), patulin (PAT), sterigmatocystin (ST) and trichothecenes-2 (T2), and the result showed that the test strip was specific to TA (Figure 6A). Moreover, to evaluate the sensitivity of the AuNF-based test strip, the TA-free standard solutions were spiked with different concentration of TA (100, 50, 25, 12.5, 6.25, 3.12, 1.56, 0.78, and 0.39 ng/ml, respectively) and determined by the constructed AuNF-based test strips. The result from Figure 6B showed that the detective threshold was 50 ng/ml, and the visual LOD was 0.78 ng/ml.

### Samples analysis of the both assays

To verify the accuracy and reproducibility of the developed LFIA in the practical detection of agricultural by-products, both apple juice and tomato ketchup samples spiked with known concentration of TA (100, 50, 25, 12.5, 6.25, 3.12, 1.56, 0.78, and 0.39 ng/ml) were used for performing the both LFIA assays. As shown in Table 1, AuNP-based LFIA assays displayed that both two kinds of spiked samples with TA concentration among 100–12.5 ng/ml had the same test results as that in the standard solution (the results from Figure 3B), demonstrating that the constructed AuNP-based strip test had good reproducibility. In addition, Table 1 proved that the AuNF-based LFIA assay results in both kinds of spiked samples with the concentrations of TA among 100–0.78 ng/ml were identical to that in the standard solution (the results from Figure 6B), while the spiked samples with the concentration of 0.39 ng/ml were hardly to visually judge the difference of color intensities between T and C lines. These results indicated that the constructed AuNF-based strip test had good accuracy and reproducibility.

**TABLE 1:** Results of visual TA detection by the AuNP-based and AuNF-based LFIA in spiked samples.

Samples	Concentration of TA spiked (ng/ml)	AuNP results (*n* = 3)	AuNF results (*n* = 3)	Verified by UHPLC-MS/MS (ng/ml)
Apple juice	100	+^a^, +, +	+, +, +	99.621 ± 0.027
	50	±^b^, ±, ±	+, +, +	49.324 ± 0.033
	25	±, ±, ±	±, ±, ±	24.379 ± 0.054
	12.5	±, ±, ±	±, ±, ±	12.462 ± 0.051
	6.25	−^c^, −, −	±, ±, ±	6.107 ± 0.052
	3.12	−, −, −	±, ±, ±	2.998 ± 0.025
	1.56	−, −, −	±, ±, ±	1.432 ± 0.043
	0.78	−, −, −	±, ±, ±	0.743 ± 0.043
	0.39	−, −, −	−^c^, −, −	0.364 ± 0.037
Tomato ketchup	100	+, +, +	+, +, +	98.946 ± 0.056
	50	±, ±, ±	+, +, +	49.279 ± 0.062
	25	±, ±, ±	±, ±, ±	24.722 ± 0.039
	12.5	±, ±, ±	±, ±, ±	12.201 ± 0.067
	6.25	−, −, −	±, ±, ±	6.128 ± 0.038
	3.12	−, −, −	±, ±, ±	3.089 ± 0.028
	1.56	−, −, −	±, ±, ±	1.481 ± 0.022
	0.78	−, −, −	±, ±, ±	0.729 ± 0.043
	0.39	−, −, −	−, −, −	0.356 ± 0.052

a No coloration at the T line, positive result.

b The color density of the T line is lighter than that of the C line; positive result.

c The color density of the T line is as the same as that of the C line, negative result.

**FIGURE 7:**
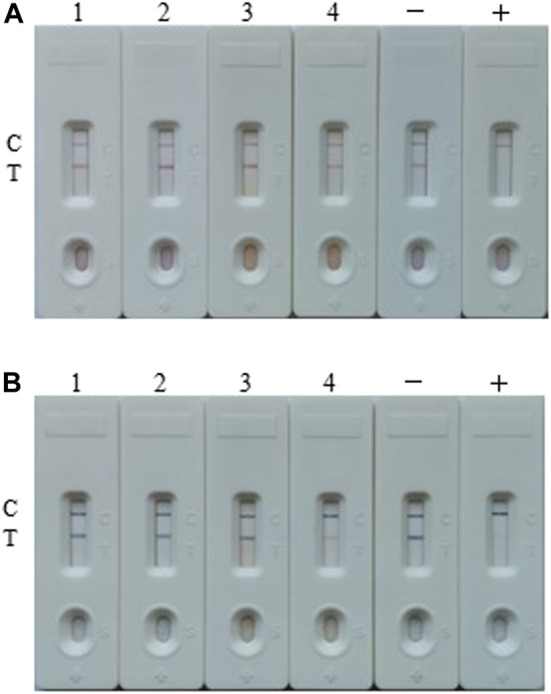
Real samples analysis of the both assays. **(A)** The real samples detection result by the AuNP-based strip. **(B)** The real samples detection results by the AuNF-based strip test. Sample 1 and 2 were apple juice, while sample 3 and 4 were tomato ketchup. Samples 1–4 contain 0.051 ± 0.043, 0.073 ± 0.041, 0.037 ± 0.032 and 5.815 ± 0.034 ng/ml TA, respectively (tested by UHPLC-MS/MS). +: positive, ‑: negative.

At last, the both developed LFIA assays were performed to determine TA in real samples, and four random real samples (samples 1 and 2 were apple juice, while samples 3 and 4 were tomato ketchup) from local super-market were tested. The results of AuNP-based assays from Figure 7A showed that TA was not detected in these four samples (the concentrations of TA in the real sample 1–4 were 0.051 ± 0.043, 0.073 ± 0.041, 0.037 ± 0.032 and 5.815 ± 0.034 ng/ml, respectively, which were determined by UHPLC-MS/MS), while the results of AuNF-based assays from Figure 7B showed that TA was detected in sample 4.

## Discussion

The contamination of mycotoxins in food products has become worldwide issue. TA was one of the strongly toxic *Alternaria* mycotoxins, which can cause human and livestock poisoning. However, there is still no available estimate of tolerable daily TA intake, so it is impossible to determine either the highest acceptable level in foodstuff or the minimum sensitivity requirements of analytical methods (Gross et al., 2011; Kong et al., 2017). In this study, the developed lateral flow assays were sufficiently sensitive to provide threshold value for TA detection. Compared with the existing LFIA for TA (Kong et al., 2017), these two kinds of lateral flow assays based on AuNP and AuNF in this study were respectively enhanced by 32 and 64 folds of threshold, and were respectively enhanced by 32 and 513 folds of visual LOD. Compared with our previous work (platinum - modified colloidal gold lateral flow) (Cai et al., 2021), this AuNF strip performed the simple one-step lateral flow procedure and shortened 2–4 folds of the assay time.

In this study, the rapid lateral flow techniques were applied to detect trace TA, and the excellent sensitivity of the technology was benefited by not only superior antibody reagent but also signal-enhanced probe. The AuNF could be used as ideal probe material to promote the sensitivity due to the localized surface plasmon resonances of complex 3D structure (Ji et al., 2015). The morphology of AuNF particle was one important character of the AuNF probe. The previous researches have shown that LFIA based on AuNF with long tip achieved the 4-fold sensitivity to that based on AuNP with similar sizes (Zhang et al., 2019). The size of AuNF particle was another important character. A recent study showed that AuNF with sizes of 47 and 79 nm showed the highest sensitivity among the five different sizes (33, 47, 79, 152, and 195 nm), and the AuNFs with 47 and 79 nm exhibited approximately 2-fold sensitivity to that of AuNF with 33 nm (Zhang et al., 2019). The AuNF with size of 75 ± 5 nm was confirmed 10-fold sensitivity to that of traditional 20 nm AuNP (Ji et al., 2015). Our previous works showed the AuNF of near 85 nm showed 4-fold sensitivity to AuNP (Ling et al., 2020), and another AuNF of near 80 nm showed 12-fold sensitivity to AuNP (Ling et al., 2021). Therefore, the sensitivity of the AuNF-based LFIA was closely related to the particle morphology and size of AuNF. In this work, the size of near 50 nm AuNF was just right in the range of 47–79 nm, consistent with the research result of Zhang and coworkers (Zhang et al., 2019).

In conclusion, to meet the urgent need to rapidly detect TA and ensure food safety, a qualitatively visual detection method of lateral flow assay based on AuNP of 17.52 nm was successfully established and used for detecting TA in agricultural by-products. The results of the AuNP-based strip assay could be visually judged by the naked eyes within 15 min, and the detective threshold for TA was 100 ng/ml, while the visual LOD was 12.5 ng/ml. To further improve the sensitivity for the detection of trace TA, the AuNP was used as seeds to produce the flower-like AuNF particle with the size near 50 nm, and another visual detection method of lateral flow assay based on the AuNF particle was further constructed and conducted for TA determining. Within 15 min, the detective threshold was 50 ng/ml and the visual LOD was 0.78 ng/ml with less matrix effect. The sensitivity of the AuNF strip was much better than that of the reported AuNP strip (Kong et al., 2017). Therefore, these two developed lateral flow assays could be used for the rapid detection of trace TA in real sample, and they were suitable for different precision requirements.

## Data Availability

The original contributions presented in the study are included in the article/Supplementary Material, further inquiries can be directed to the corresponding author.

## References

[B1] Anfossi L. Di Nardo F. Giovannoli C. Passini C. Baggiani C. (2013). Increased sensitivity of lateral flow immunoassay for ochratoxin A through silver enhancement. Anal. Bioanal. Chem. 405, 9859–9867. 10.1007/s00216-013-7428-6 24162821

[B2] Ang S. H. Yu C. Y. Ang G. Y. Chan Y. Y. Alias Y. B. Khor S. M. (2015). A colloidal gold-based lateral flow immunoassay for direct determination of haemoglobin A1c in whole blood. Anal. Methods 7, 3972–3980. 10.1039/c5ay00518c

[B4] Asam S. Rychlik M. (2013). Potential health hazards due to the occurrence of the mycotoxin tenuazonic acid in infant food. Eur. Food Res. Technol. 236, 491–497. 10.1007/s00217-012-1901-x

[B3] Asam S. Liu Y. Konitzer K. Rychlik M. (2011). Development of a stable isotope dilution assay for tenuazonic acid. J. Agric. Food Chem. 59, 2980–2987. 10.1021/jf104270e 21370870

[B5] Cai P. Wang R. Ling S. Wang S. (2021). A high sensitive platinum-modified colloidal gold immunoassay for tenuazonic acid detection based on monoclonal IgG. Food Chem. x. 360, 130021. 10.1016/j.foodchem.2021.130021 33991976

[B6] Chenggang S. H. I. Suqing Z. Kun Z. Guobao H. Zhenyu Z. H. U. (2008). Preparation of colloidal gold immunochromatography strip for detection of methamidophos residue. J. Environ. Sci. 20, 1392–1397. 10.1016/S1001-0742(08)62238-X 19202881

[B7] Dondapati S. K. Sau T. K. Hrelescu C. Klar T. A. Stefani F. D. Feldmann J. (2010). Label-free biosensing based on single gold nanostars as plasmonic transducers. ACS Nano 4, 6318–6322. 10.1021/nn100760f 20942444

[B8] Eskola M. Kos G. Elliott C. T. Hajšlová J. Mayar S. Krska R. (2020). Worldwide contamination of food-crops with mycotoxins: Validity of the widely cited ‘FAO estimate’ of 25%. Crit. Rev. Food Sci. Nutr. 60, 2773–2789. 10.1080/10408398.2019.1658570 31478403

[B9] Giambrone J. J. Davis N. D. Diener U. L. (1978). Effect of tenuazonic acid on young chickens. Poult. Sci. 57, 1554–1558. 10.3382/ps.0571554 751036

[B10] Griffin G. F. Chu F. S. (1983). Toxicity of the Alternaria metabolites alternariol, alternariol methyl ether, altenuene, and tenuazonic acid in the chicken embryo assay. Appl. Environ. Microbiol. 46, 1420–1422. 10.1128/aem.46.6.1420-1422.1983 6686430 PMC239585

[B11] Gross M. Curtui V. Ackermann Y. Latif H. Usleber E. (2011). Enzyme immunoassay for tenuazonic acid in apple and tomato products. J. Agric. Food Chem. 59, 12317–12322. 10.1021/jf203540y 22054343

[B12] Hickert S. Krug I. Cramer B. Humpf H. U. (2015). Detection and quantitative analysis of the non-cytotoxic allo-tenuazonic acid in tomato products by stable isotope dilution HPLC-MS/MS. J. Agric. Food Chem. 63, 10879–10884. 10.1021/acs.jafc.5b04812 26633086

[B13] Hong X. Mao Y. Yang C. Liu Z. Li M. Du D. (2020). Contamination of zearalenone from China in 2019 by a visual and digitized immunochromatographic assay. Toxins (Basel) 12, 521. 10.3390/toxins12080521 PMC7472730 32823857

[B14] Ji Y. Ren M. Li Y. Huang Z. Shu M. Yang H. (2015). Detection of aflatoxin B1 with immunochromatographic test strips: Enhanced signal sensitivity using gold nanoflowers. Talanta 142, 206–212. 10.1016/j.talanta.2015.04.048 26003713

[B15] Kong D. Liu L. Song S. Zheng Q. Wu X. Kuang H. (2017). Rapid detection of tenuazonic acid in cereal and fruit juice using a lateral-flow immunochromatographic assay strip. Food Agric. Immunol. 28, 1293–1303. 10.1080/09540105.2017.1337085

[B16] Liang Y. F. Zhou X. W. Wang F. Shen Y. D. Xiao Z. L. Zhang S. W. (2020). Development of a monoclonal antibody-based ELISA for the detection of Alternaria mycotoxin tenuazonic acid in food samples. Food Anal. Methods 13, 1594–1602. 10.1007/s12161-020-01780-w

[B17] Ling S. Chen Q. A. Zhang Y. Wang R. Jin N. Pang J. (2015). Development of ELISA and colloidal gold immunoassay for tetrodotoxin detetcion based on monoclonal antibody. Biosens. Bioelectron. X. 71, 256–260. 10.1016/j.bios.2015.04.049 25913446

[B18] Ling S. Li X. Zhao Q. Wang R. Tan T. Wang S. (2020). Preparation of monoclonal antibody against penicillic acid (PA) and its application in the immunological detection. Food Chem. x. 319, 126505. 10.1016/j.foodchem.2020.126505 32169762

[B19] Ling S. Xiao S. Xie C. Wang R. Zeng L. Wang K. (2018). Preparation of monoclonal antibody for brevetoxin 1 and development of Ic-ELISA and colloidal gold strip to detect brevetoxin 1. Toxins (Basel) 10, 75–11. 10.3390/toxins10020075 PMC5848176 29419743

[B20] Ling S. Zhao Q. Iqbal M. N. Dong M. Li X. Lin M. (2021). Development of immunoassay methods based on monoclonal antibody and its application in the determination of cadmium ion. J. Hazard. Mat. 411, 124992. 10.1016/j.jhazmat.2020.124992 33454572

[B21] Lohrey L. Marschik S. Cramer B. Humpf H. U. (2013). Large-scale synthesis of isotopically labeled 13C 2-tenuazonic acid and development of a rapid HPLC-MS/MS method for the analysis of tenuazonic acid in tomato and pepper products. J. Agric. Food Chem. 61, 114–120. 10.1021/jf305138k 23230907

[B22] López-Marzo A. M. Pons J. Blake D. A. Merkoçi A. (2013). High sensitive gold-nanoparticle based lateral flow Immunodevice for Cd2+ detection in drinking waters. Biosens. Bioelectron. X. 47, 190–198. 10.1016/j.bios.2013.02.031 23578973

[B23] Ostry V. (2008). Alternaria mycotoxins : An overview of chemical characterization , producers , toxicity , analysis and occurrence in foodstuffs. World Mycotoxin J. 1, 175–188. 10.3920/wmj2008.x013

[B24] Panigrahi S. Dallin S. (1994). Toxicity of the Alternaria spp metabolites, tenuazonic acid, alternariol, altertoxin‐i, and alternariol monomethyl ether to brine shrimp (*Artemia salina* L) larvae. J. Sci. Food Agric. 66, 493–496. 10.1002/jsfa.2740660411

[B25] Puntscher H. Kütt M. Skrinjar P. Mikula H. Podlech J. Fröhlich J. (2018). Tracking emerging mycotoxins in food : Development of an LC-MS/MS method for free and modified Alternaria toxins, 4481–4494. 10.1007/s00216-018-1105-8 PMC6021461 29766221

[B26] Saeed A. F. U. H. Ling S. Yuan J. Wang S. (2017). The preparation and identification of a monoclonal antibody against domoic acid and establishment of detection by indirect competitive ELISA. Toxins (Basel) 9, 250. 10.3390/toxins9080250 PMC5577584 28817087

[B27] Siegel D. Merkel S. Koch M. Nehls I. (2010). Quantification of the Alternaria mycotoxin tenuazonic acid in beer. Food Chem. x. 120, 902–906. 10.1016/j.foodchem.2009.10.070 19941132

[B28] Siegel D. Rasenko T. Koch M. Nehls I. (2009). Determination of the Alternaria mycotoxin tenuazonic acid in cereals by high-performance liquid chromatography–electrospray ionization ion-trap multistage mass spectrometry after derivatization with 2, 4-dinitrophenylhydrazine. J. Chromatogr. A 1216, 4582–4588. 10.1016/j.chroma.2009.03.063 19361805

[B29] Wang F. Li Z. F. Wan D. B. Vasylieva N. Shen Y. D. Xu Z. L. (2021a). Enhanced non-toxic immunodetection of Alternaria mycotoxin tenuazonic acid based on ferritin-displayed anti-idiotypic nanobody-nanoluciferase multimers. J. Agric. Food Chem. 69, 4911–4917. 10.1021/acs.jafc.1c01128 33870684

[B30] Wang R. Wang J. Liu H. Gao Y. Zhao Q. Ling S. (2021b). Sensitive immunoassays based on speci fi c monoclonal IgG for determination of bovine lactoferrin in cow milk samples. Food Chem. x. 338, 127820. 10.1016/j.foodchem.2020.127820 32827899

[B31] Wang R. Zeng L. Yang H. Zhong Y. Wang J. Ling S. (2017). Detection of okadaic acid (OA) using ELISA and colloidal gold immunoassay based on monoclonal antibody. J. Hazard. Mat. 339, 154–160. 10.1016/j.jhazmat.2017.06.030 28648727

[B32] Wu Y. Zhou Y. Leng Y. Lai W. Huang X. Xiong Y. (2020). Emerging design strategies for constructing multiplex lateral flow test strip sensors. Biosens. Bioelectron. X. 157, 112168. 10.1016/j.bios.2020.112168 32250938

[B33] Yan L. Dou L. Bu T. Huang Q. Wang R. Yang Q. (2018). Highly sensitive furazolidone monitoring in milk by a signal amplified lateral flow assay based on magnetite nanoparticles labeled dual-probe. Food Chem. x. 261, 131–138. 10.1016/j.foodchem.2018.04.016 29739573

[B34] Yekeler H. Bitmiş K. Özçelik N. Doymaz M. Z. Çalta M. (2001). Analysis of toxic effects of Alternaria toxins on esophagus of mice by light and electron microscopy. Toxicol. Pathol. 29, 492–497. 10.1080/01926230152499980 11560255

[B35] Zangheri M. Cevenini L. Anfossi L. Baggiani C. Simoni P. Di F. (2015). A simple and compact smartphone accessory for quantitative chemiluminescence-based lateral flow immunoassay for salivary cortisol detection. Biosens. Bioelectron. X. 64, 63–68. 10.1016/j.bios.2014.08.048 25194797

[B36] Zhang W. Duan H. Chen R. Ma T. Zeng L. Leng Y. (2019). Effect of different-sized gold nanoflowers on the detection performance of immunochromatographic assay for human chorionic gonadotropin detection. Talanta 194, 604–610. 10.1016/j.talanta.2018.10.080 30609579

[B37] Zhou B. Qiang S. (2008). Environmental, genetic and cellular toxicity of tenuazonic acid isolated from Alternaira alternata. Afr. J. Biotechnol. 7, 1151–1156.

